# EEG Recorded from the Ear: Characterizing the Ear-EEG Method

**DOI:** 10.3389/fnins.2015.00438

**Published:** 2015-11-18

**Authors:** Kaare B. Mikkelsen, Simon L. Kappel, Danilo P. Mandic, Preben Kidmose

**Affiliations:** ^1^Department of Engineering, Aarhus UniversityAarhus, Denmark; ^2^Department of Electrical and Electronic Engineering, Imperial CollegeLondon, UK

**Keywords:** ear-EEG, mobile EEG, auditory evoked potentials, auditory steady-state response, alpha band power

## Abstract

**Highlights**

- Auditory middle and late latency responses can be recorded reliably from ear-EEG.
- For sources close to the ear, ear-EEG has the same signal-to-noise-ratio as scalp.
- Ear-EEG is an excellent match for power spectrum-based analysis.

A method for measuring electroencephalograms (EEG) from the outer ear, so-called ear-EEG, has recently been proposed. The method could potentially enable robust recording of EEG in natural environments. The objective of this study was to substantiate the ear-EEG method by using a larger population of subjects and several paradigms. For rigor, we considered simultaneous scalp and ear-EEG recordings with common reference. More precisely, 32 conventional scalp electrodes and 12 ear electrodes allowed a thorough comparison between conventional and ear electrodes, testing several different placements of references. The paradigms probed auditory onset response, mismatch negativity, auditory steady-state response and alpha power attenuation. By comparing event related potential (ERP) waveforms from the mismatch response paradigm, the signal measured from the ear electrodes was found to reflect the same cortical activity as that from nearby scalp electrodes. It was also found that referencing the ear-EEG electrodes to another within-ear electrode affects the time-domain recorded waveform (relative to scalp recordings), but not the timing of individual components. It was furthermore found that auditory steady-state responses and alpha-band modulation were measured reliably with the ear-EEG modality. Finally, our findings showed that the auditory mismatch response was difficult to monitor with the ear-EEG. We conclude that ear-EEG yields similar performance as conventional EEG for spectrogram-based analysis, similar timing of ERP components, and equal signal strength for sources close to the ear. Ear-EEG can reliably measure activity from regions of the cortex which are located close to the ears, especially in paradigms employing frequency-domain analyses.

## 1. Introduction

Electroencephalography (EEG) is a well established technique (Pravdich-Neminsky, 1913), providing valuable insights into brain activity, with applications both in clinical practice and in basic and applied neuroscience (Nunez and Srinivasan, 2007).

Despite the widespread adoption, a number of important applications of EEG are prohibited by the requirements for mobility and discreetness of the EEG equipment used. Examples include brain-computer interfaces (BCI), long-term monitoring of neurological patients and sleep monitoring. To this end, several EEG recording methods have recently been proposed (Casson et al., 2010; Debener et al., 2012), including some which benefit from a relatively unrestricted access to the area around and inside the ears (Looney et al., 2012; Bleichner et al., 2015; Norton et al., 2015). A very promising solution, belonging to the latter class, is the ear-EEG platform (Looney et al., 2012; Kidmose et al., 2013) which promises a robust, unobtrusive and non-invasive means for monitoring the brain activity outside the laboratory, both within and outside a clinical context.

While previous publications have presented various early demonstrations of the feasibility of ear-EEG, the objective of the current study is to conduct a rigorous and comprehensive study over a larger population of subjects and spanning several paradigms, and in this way assess the utility of ear-EEG compared to standard scalp-EEG. Our aim was also to provide a thorough and self-sufficient reference for practitioners, regarding the feasibility of the ear-EEG method for the wider multi-disciplinary EEG community.

## 2. Materials and methods

### 2.1. The ear EEG platform

**Figure 1:**
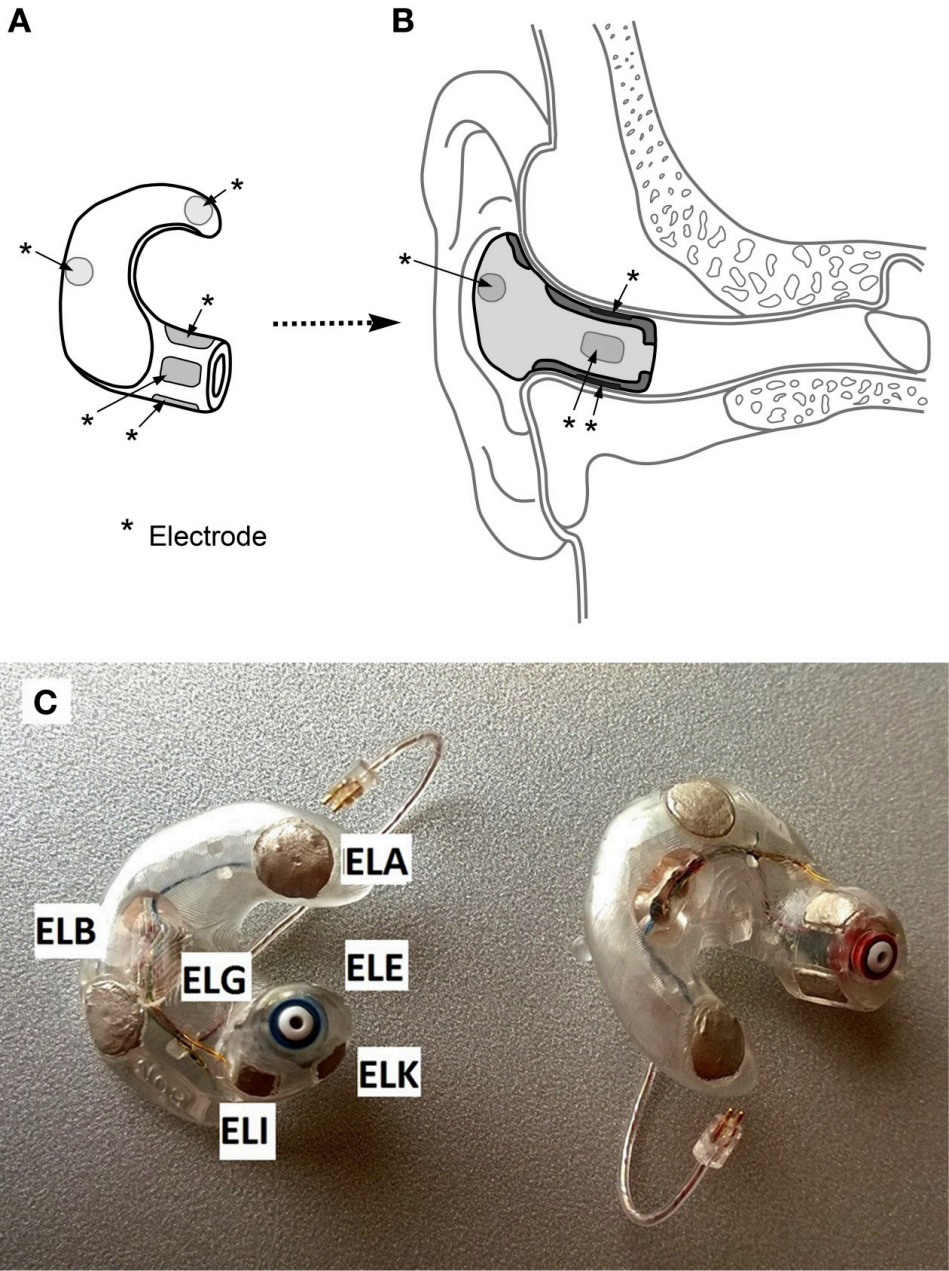
**(A)** Concept drawing of an ear-piece with electrodes embedded on the surface. Electrodes are indicated with ^*^-labels. **(B)** Cross-sectional view of an ear-piece placed in the ear. **(C)** Example of an actual ear-piece used in the study. The electrode labels are given for the left ear plug. ELG is located at the posterior surface of the ear canal, while ELE is located at the superior surface.

The ear-EEG platform comprises a set of electrodes placed inside each ear canal, together with additional electrodes in the concha of each ear. Figure 1 shows a schematic diagram and a photograph of the ear plugs with the embedded electrodes and electrode labels.

The labeling convention of the ear-EEG electrodes is E*xy*, where *x* denotes the left (L) or right (R) ear, and *y* the position within the denoted ear. Thus, the two concha electrodes are E*x*A (farthest from the ear canal) and E*x*B (closest), while the ear canal electrodes are named E*x*E, E*x*G, E*x*I, E*x*K, starting from the top of the ear canal and cycling dorsally. Additionally, see Kidmose et al. (2012) for a detailed account of the naming scheme.

In all the recordings, the ERB electrode was used as a ground electrode for both ears, and all ear electrodes were referenced to a passive electrode placed next to the scalp Cz electrode (according to the 10–20 system). The separation of the scalp and ear setups will be further commented upon in the Section 4 below.

The electrodes were connected to a g.tec USBamp amplifier, and data was collected using the g.Recorder software. The electrodes were coated in a high viscosity conductive gel (Ten20 EEG Paste), and additional gel was applied to the concha electrodes after insertion (GAMMAgel by g.tec).

In this study, we have exclusively focused on custom-made ear pieces (created using ear imprints and 3D-printing), fitted individually to each participant. While preliminary work has taken place regarding generic ear pieces (Kidmose et al., 2013; Goverdovsky et al., 2015), we have in this study focused on the best-fit scenario, which so far continues to be delivered by the custom fit solution.

### 2.2. Scalp EEG setup

Simultaneously with the ear-EEG recording, conventional 32-channel scalp EEG was recorded, using a g.tec EEG cap with active electrodes, all Cz-referenced. The 32 scalp electrodes were named according to the 10–20 system, and were: C1, C2, C3, C4, C5, C6, CP3, CP4, CP5, CP6, F7, F8, FC3, FC4, FC5, FC6, FCz, FT7, FT8, Fz, P3, P4, P5, P6, P7, P8, T7, T8, TP10, TP7, TP8, TP9. Whenever scalp data is used, we will make it clear which channels are relevant (primarily Cz and those close to the ears).

### 2.3. Data collection

The pool of volunteer subjects who participated in this study consisted of 13 individuals, aged 23–43, median 30, five of which were female, all right handed. The study was approved by the regional scientific ethics committee, and the national Danish Health and Medicines Authority^1^. As per the guidelines of these authorities, all participants were given written and oral information, and all gave written, informed consent before participating, after having had ample time for consideration.

Prior to insertion of the ear plugs, the ears were cleaned with ethanol and abrasive gel. After insertion, the impedances of all the electrodes relative to the ground electrode were measured using g.Recorder. If more than two electrode impedances within one ear were above 10 k℧, the cleaning of that ear was repeated, once.

Of the described paradigms (below), the auditory steady-state response (ASSR) paradigm was recorded first, and served as an additional check of electrode connections, before performing the remaining paradigms. Where required, the cleaning of the ear canal and concha was repeated, as dictated by the quality of the ASSR recording.

Due to equipment failure, only 10 of the 13 subjects were used in the α-attenuation paradigm.

### 2.4. Paradigms

Before every auditory stimulus, the sound amplitude was adjusted to the maximum of what the subject thought s/he was comfortable with for the duration of the stimulus.

The participants were subjected to three different paradigms, described below:

**Auditory Steady-State Response (ASSR)**. The auditory steady-state response was evoked using amplitude modulated stationary white noise. The amplitude modulation frequency was 40 Hz, and the stimulus was presented binaurally for 4 min. The ASSR is created because the cochlear performs a frequency specific encoding of the signal amplitude, such that the neural encoding reflects the amplitude modulation, and as a result the amplitude modulation frequency is observable in the frequency spectrum of the EEG (Galambos et al., 1981). The power spectrum was calculated by averaging 240 1-s intervals, aligned using a 20 Hz trigger. The signal was sampled at 256 Hz. It is important to mention that the ASSR has a wide scope, as the ASSR is a clear, easy to use indicator of whether the signal is neural in origin.

**Mismatch Negativity (MMN) paradigm**. A well established stimulus is the so-called oddball paradigm, which was used here to test mismatch negativity (MMN; Näätänen et al., 1978). For ease of analysis, we used a well known and optimized oddball paradigm, introduced in Näätänen et al. (2004), whereby every other stimuli was a standard beep consisting of a mixture of sinusoidal tones of 500, 1000, and 1500 Hz, lasting 75 ms. The remaining stimuli were oddballs in the sense of low/high pitch, low/high volume, left/right delay, reduced duration or having a gap. Following Näätänen et al. (2004), the measurements were grouped in sessions of 1845 stimuli, each session lasting 15 min and 20 s. The sessions were structured as three repetitions of subsessions, each subsession consisting of 15 standard stimuli followed by 600 alternating standard and oddball stimuli. Between the sessions were short breaks in which the subjects were asked about their comfort. Each subject completed 4 sessions, totalling 7380 stimuli. The 15 standards at the start of each subsession were discarded in the subsequent analysis, bringing each per-subject data set down to 7200 stimuli.

During measurements, the subject was watching a silent movie of their own choice, without subtitles.

α**-attenuation paradigm**. The subjects were instructed alternately to rest with closed eyes, and doing simple arithmetic in the head with open eyes. More precisely, the subjects were shown numbers from the interval 50–100, which changed every 10 s, and tasked with repeatedly subtracting 7 from the number. Using auditory cues appearing at 1 min intervals, the subjects alternated between performing eyes-open arithmetic and relaxing with eyes closed.

### 2.5. Data preprocessing

The first step was to split the 32 scalp channels and 11 ear channels into separate datasets. As the ear-EEGs were Cz-referenced during recording, a “proper” ear-EEG dataset was obtained by rereferencing to the ExA-electrodes, see Figure 1. Furthermore, “cross-referenced” datasets were created by referencing each ear-EEG channel to the average of all non-discarded ear-channels in the opposite ear. These different reference-setups are intended to investigate the possibilities of different hardware setups in the final ear-EEG platform, for instance comparing a single ear piece with a setup in which two pieces are physically connected.

Electrode channels were rejected based on 2 criteria. We list the rejection rates for the E*x*A-referenced dataset along with the descriptions:

1. Channels which did not have a clear 40 Hz peak in the ASSR-measurements were discarded and do not appear in the rest of the analysis. The precise criterion for “clear” was a 9 dB amplitude-difference relative to surrounding noise floor, a value chosen because it most cleanly partitions the distribution of measured amplitude-peak-heights in distinct sets of high and low values. This accounts for a channel rejection rate of 7.7% in the E*x*A-referenced dataset.
2. Second, channels for which Cz-referenced mismatch responses in ear electrodes did not have shapes similar to what was recorded in Cz-referenced TP9 and TP10 electrodes (both of which are placed close to the ears) were discarded. It was made sure that this criterion never resulted in rejection of entire ears, which could have resulted in a bias in the analysis toward subjects with similar ERPs. This criterion accounts for a channel rejection rate of 9.3% in the E*x*A-referenced dataset.

In total, the channel rejection rate is 17%. By going over the numbers, it appears that many of the 17% are due to unstable skin-to-electrode connections at the E*x*A-electrode, which after rereferencing affects the rest of the ear-electrodes. Thus, it is probable that a better connection at the E*x*A-electrode would significantly bring down the rejection rate. It would also be possible to reference each electrode to the average signal within each ear; however given the relatively low number of electrodes in each ear, this would mean that an ear piece with one or two rejected electrodes would have a much differently weighted average than a full piece, causing unwanted problems when comparing ERPs. By sticking with a single-electrode reference, we have made sure that all non-rejected electrodes are as equivalent as possible, across subjects.

The preprocessing was performed in Matlab, using a combination of EEGLAB (Delorme and Makeig, 2004), ERPLAB (Lopez-Calderon and Luck, 2014) and custom scripts.

#### 2.5.1. Mismatch negativity (MMN) paradigm

Irregularities in the measured triggers were observed in two MMN sessions (1845 stimuli), making up 3.8% of the whole. These sessions were removed due to this before the data analysis.

Each channel was subjected to a bandpass filter, using the FIR filter in EEGLAB with pass band 3-30 Hz. Subsequently, the data was partitioned into epochs of [-100 ms, 600 ms] relative to each stimulus onset, and every epoch was baseline corrected according to the [-100 ms, 0] interval. After baseline correction, the epochs were rejected if the absolute value in any channel exceeded 100 mV for Cz- and cross-referenced data, and 50 mV for ExA-referenced data, resulting in an average rejection rate of 0.7%. Finally, epochs were trend-corrected if their straight line fit had *R*^2^ > 0.2, and were then baseline corrected again, *R*^2^ being the well-known goodness-of-fit measure.

#### 2.5.2. α-attenuation paradigm

The pass band in this case was 0.5–45 Hz, and epochs, of 4 s duration, were discarded based on the same amplitude criteria used for the MMN paradigm. The epoch rejection rate was 9.6%. The difference in rejection rates is most likely due to the different pass bands used.

## 3. Results

### 3.1. Cz-referenced ERP

**Figure 2:**
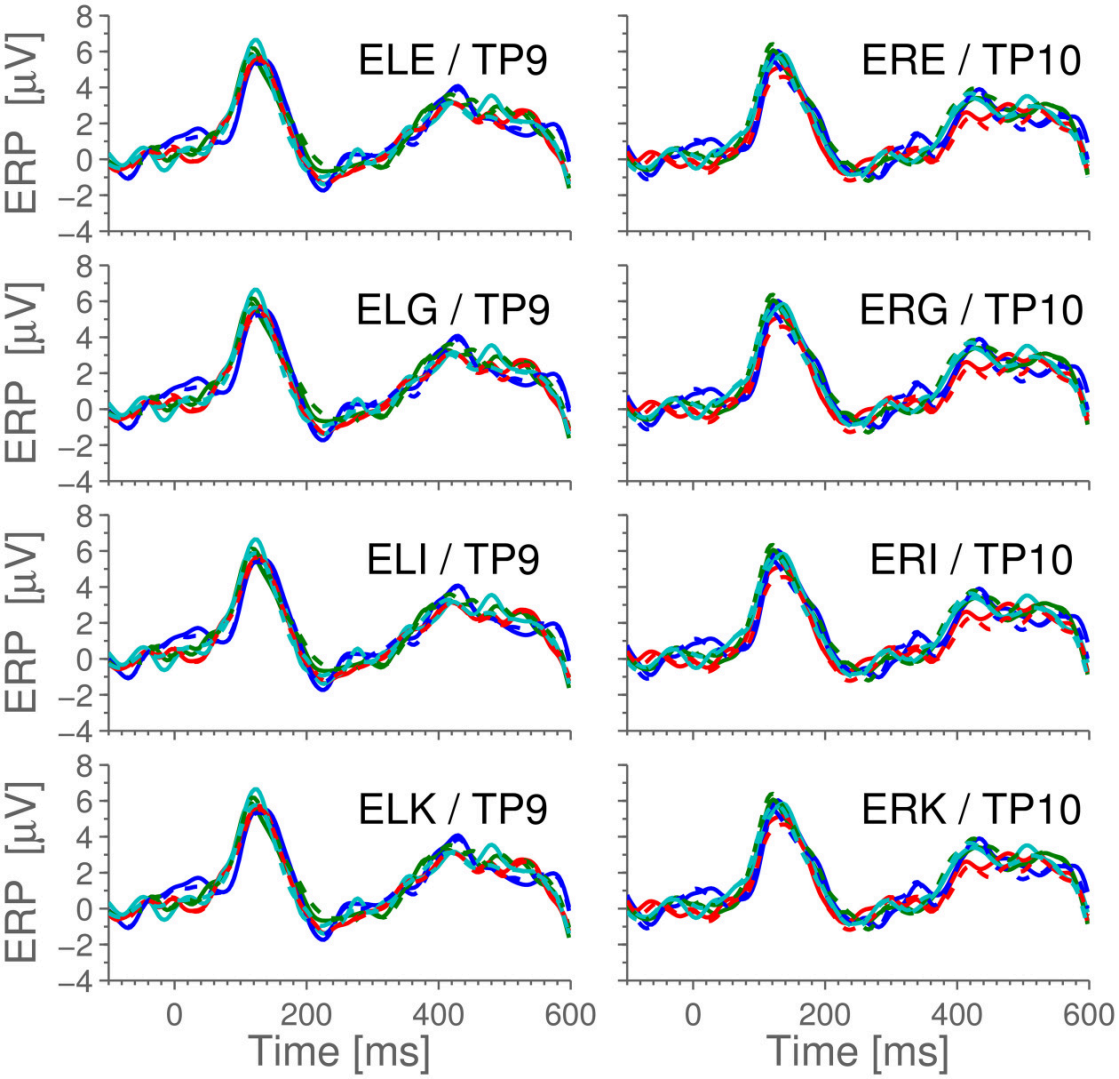
**The difference-waveforms (showing the mismatch response to oddballs) for scalp and ear-electrode ERPs, with reference in Cz**. The data shown is example data from a single, representative subject. Different colors correspond to different sessions of 1800 epochs, while scalp and ear-ERPs from the same session have identical colors. Full lines are scalp data, dashed lines are ear-data. Observe that the difference waves from the two electrode types are very similar.

The Cz-referenced ear data were used to investigate the relationship between scalp and ear-EEG; notice that owing to the on-scalp reference, the ear electrodes essentially become just additional scalp electrodes. Figure 2 shows an example plot of the difference waveforms (difference between standards and pooled oddballs in the MMN paradigm) from a single subject, where different colors correspond to different sessions, and ear and scalp electrodes are in the same colors. By visual inspection, we observe that the scalp- and ear-ERPs are very similar, with an average correlation coefficient between scalp and ear of 0.977 (for the data shown, see below for the whole population). As scalp potentials are spatially low-pass filtered by the transfer function from the neural sources to the surface of the head, small changes in electrode positions do not give rise to dramatic changes in the measured potential. Therefore, considering that the distance to the reference electrode (Cz) was large compared to the distance between the measuring electrodes, it is not surprising that the potentials measured from the ear electrodes were essentially the same as the potentials measured from nearby temporal region scalp electrodes. This confirmation, that the signal measured from the ear electrodes reflects the same neuronal activity as that on the scalp, validates the ear electrodes as an equally reliable and faithful means for recording brain activity as the scalp electrodes, when the reference is the same.

Across the entire dataset (44 trials), the correlation between simultaneous scalp and ear-recordings is 0.96 ± 0.03, and the variation between sessions is larger than between electrode types. It is worth noting that the observed 150 ms peak is in perfect agreement with the literature (Näätänen et al., 2004), thus demonstrating that the MMN paradigm is observable from the ear-electrodes when referenced to a scalp-electrode.

**Figure 3:**
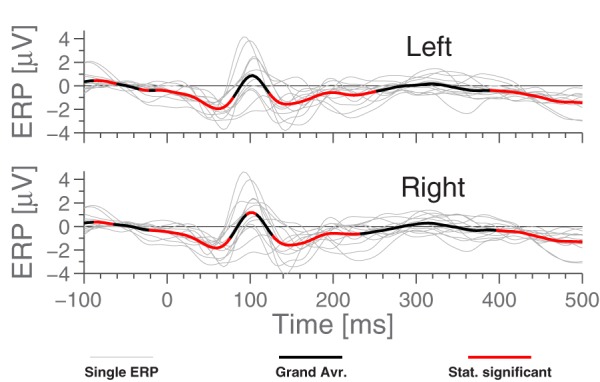
**The ERPs for the subjects in response to the standard stimulus and the grand average, Cz-referenced**. The ERPs have been averaged across electrodes within each ear, and the employed preprocessing was as explained in the Section 2.5 Data Processing. Highlighted portions of the grand average are significantly different from 0 according to a *t*-test with significance level 5%.

For comparison with later figures, Figure 3 shows the ERP for standard stimulus averaged within each ear, referenced to Cz.

### 3.2. ASSR

Having verified that the ear electrodes exhibit the required electrical properties (Figure 2), the ear electrodes were next referenced to the ExA electrode of the same ear, thereby mimicking an independent ear-EEG setup (as described in Section 2.5).

**Figure 4:**
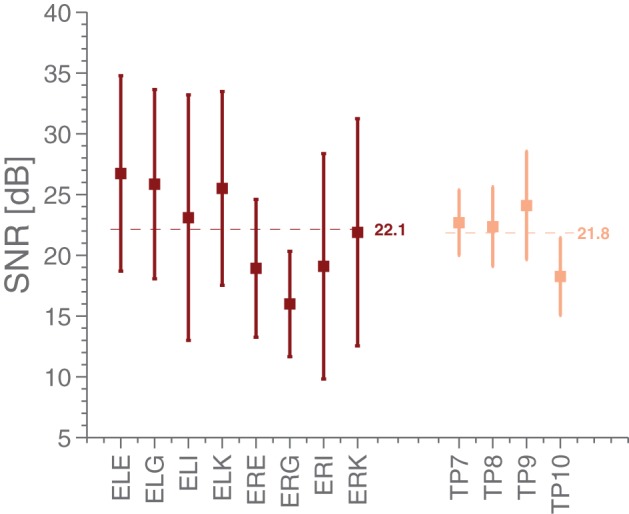
**The mean and standard deviations of SNR estimates in the ASSR paradigm for various EEG electrodes**. The ear electrodes are referenced to the A-electrode in the same ear, while the scalp electrodes are referenced to Cz. The error bars for each estimate represent the standard deviation.

**Figure 5:**
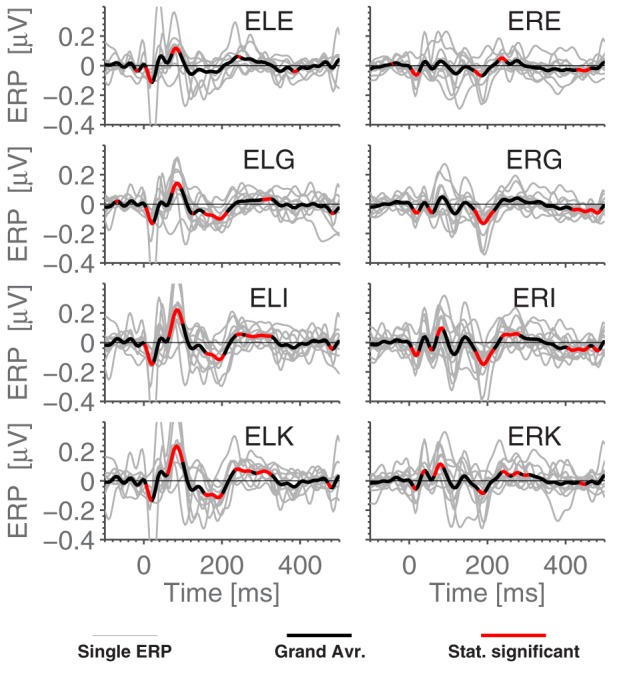
**The ERPs for the subjects in response to the standard stimulus and the grand average, ExA-referenced**. Subplot titles refer to electrode labels, and the employed preprocessing was as explained in the Section 2.5 Data Processing. Highlighted portions of the grand average are significantly different from 0 according to a *t*-test with significance level 5%.

Using the ASSR paradigm, we then estimated the signal-to-noise ratios (SNR) for both scalp and ear-EEG setups, whereby the SNR was defined as the difference between the logarithm of the power at 40 Hz (the signal) and the logarithm of the average power in 5 Hz intervals around 40 Hz (the noise floor). Figure 4 shows statistics of the ASSR SNR in each ear-EEG channel and for four nearby scalp channels. Generally, the SNR in non-discarded ear-EEG channels was 22.1 ± 8.5, while for TP9/TP10 it was 21.8 ± 4.0, after averaging 240 segments. The scalp electrodes were referenced to Cz. It is interesting to note the difference in SNR between left and right ear, as can be seen in Figure 4. This is likely related to the left-right differences observed in Figure 5.

### 3.3. Ear referenced ERPs

Figure 5 shows the grand average ERPs for the standard stimuli in the MMN paradigm, where each subject-line (thin, light gray) represents the averaged ERP for a single subject (average over 7200 epochs). The grand average, plotted in bold black, was calculated as a simple arithmetic mean over all single subject ERPs. Some difference can be observed between the ears, but very little within each ear. More precisely, the calculated correlation coefficient between sessions (1800 stimuli) on the same person averages to 0.45, while using the set of 7200 stimuli, we find 0.80 between EEG channels in the same ear and 0.75 between ear averages.

**Figure 6:**
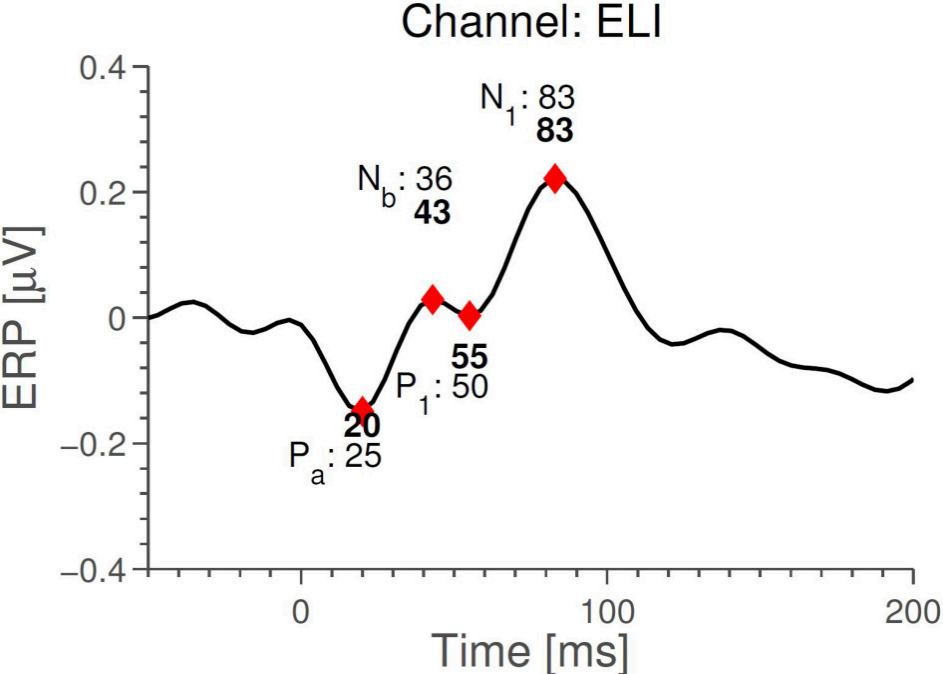
**Comparison between timings of middle latency AEPs measured with the ear-EEG platform (ExA-reference) for the standard stimulus and those reported in Picton et al. (1974)**. Timings in bold are those measured with ear-EEG. The precision is 4 ms, corresponding to the time difference between measurements.

We next compared timings of the middle latency deflections in the grand average with those reported by Picton et al. (1974). Figure 6 shows that the timings of the early to medium latency AEPs (Auditory Evoked Potentials) are close to the previously reported latencies, especially considering that the sample rate of 256 Hz only allows a timing precision of 4 ms. It seems plausible that the observed timings of *N*_*b*_ and *P*_1_ depend on electrode positions, since the contributions to the signal from those components overlap. We find that the polarities of the potentials, for the considered electrodes, are opposite to those reported in Picton et al. (1974) (so N_*b*_ and N_1_ are positive deflections). Given that in the ear-EEG setup, both electrodes (active and reference) were moved compared to Picton et al. (1974), this is not cause for concern. The N_1_ response is clearest in the left ear, possibly explaining the difference between the ears seen in Figure 4. We relate these differences to the known interhemispheric differences in auditory cortex, both functionally (Shtyrov et al., 1999; Rodrigoet al., 2008) and anatomically (Penhune et al., 1996; Dorsaint-Pierre et al., 2006).

Comparing Figures 3 and 5, we see that while *N*_1_ is present in both ERPs, they are largely different otherwise. In particular, the Cz-referenced ERP does not have the same level of detail in the early parts of the ERP (before ≈70 ms).

**Figure 7:**
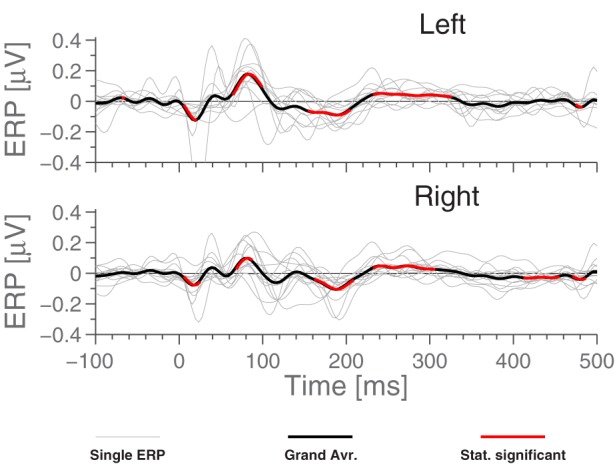
**ERP for aggregate ear canals (averaging E,I,K and G electrodes of each ear) for standard stimulus, each referenced to the local ExA-electrode**. Light gray lines represent individual subjects. Highlighted portions of the grand average are significantly different from 0 according to a *t*-test with significance level 5%.

Based on Figure 5, it is natural to ask how the observed significance levels change if the recordings are averaged within each ear, to form just two “aggregate” electrodes, one for each ear. This is shown in Figure 7, where it is observed that, not surprisingly, that the shape of the ERPs are different from those shown in Figure 5.

The analysis for the MMN paradigm (average oddball response minus standard response) did not reveal any significant MMN response, when using this electrode setup. In other words, the grand average was not observed to be meaningfully different from 0. Therefore, we have decided not to dwell further on the analysis of this part of our observations.

Finally, analysis of the “cross-referenced” setup revealed a slight increase in ERP amplitude, as should be expected. However, the ERPs do not contribute any additional features or offer an improvement to measurement significance compared to Figure 7. This is most probably due to the symmetric nature of the paradigm. Due to this, we have not focused further on this dataset.

### 3.4. Same ear referenced alpha power

**Figure 8:**
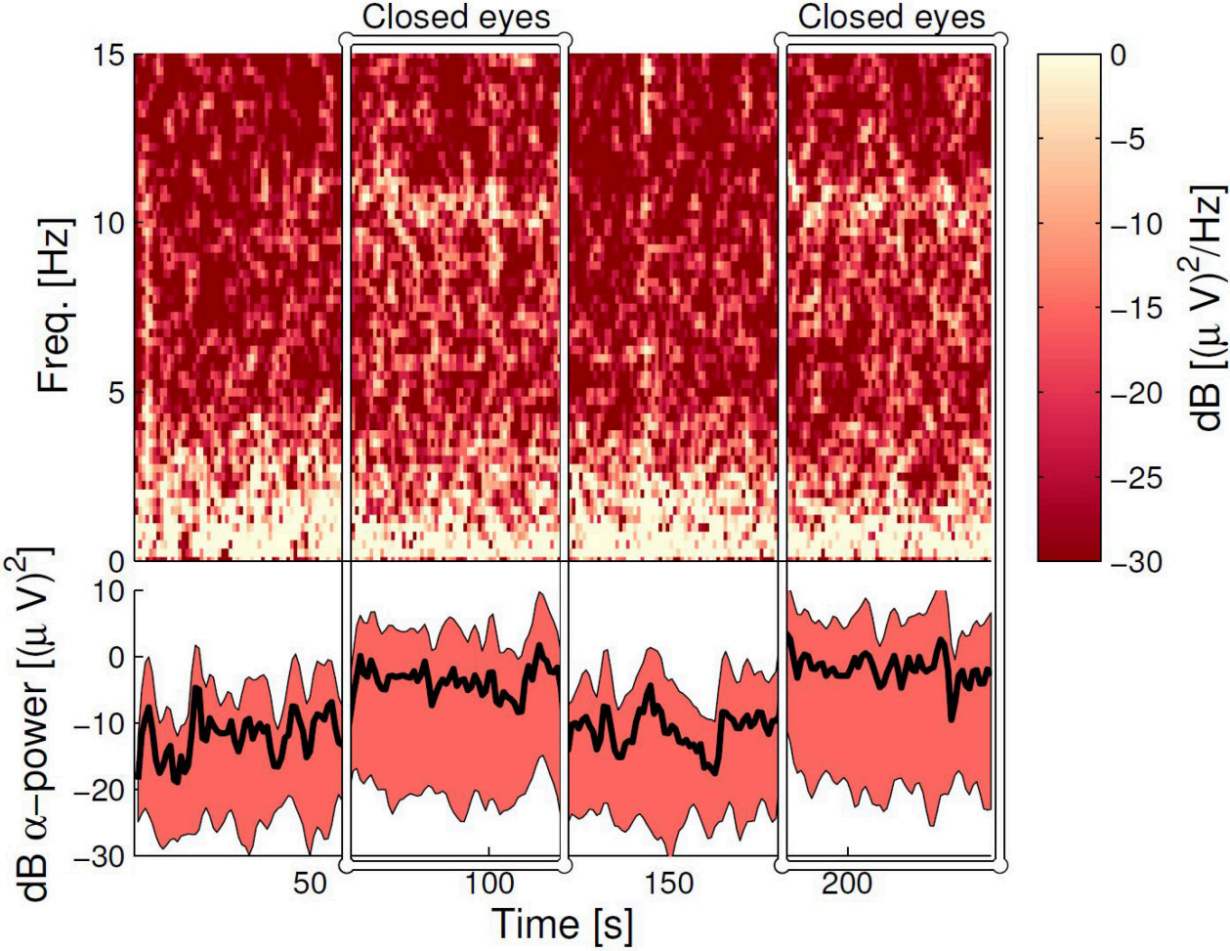
**The α-band power during the α-attenuation paradigm for the ELE channel**. **Top panel:** the spectrogram for a specific subject, where each column shows the power spectrum for a 4-s data segment. Neighboring columns have time offset by 1 s (meaning 3 s overlap). **Bottom panel:** Averaged power in the 8–12 Hz band, averaged over all subjects. The wide band demarcates the area between 15 and 85% percentiles, and has been smoothed with a moving average filter of 5 s width. The line within the band shows the grand average, not smoothed. In both panels, the dB scale is relative to 1 (µV)^2^/Hz.

**Figure 9:**
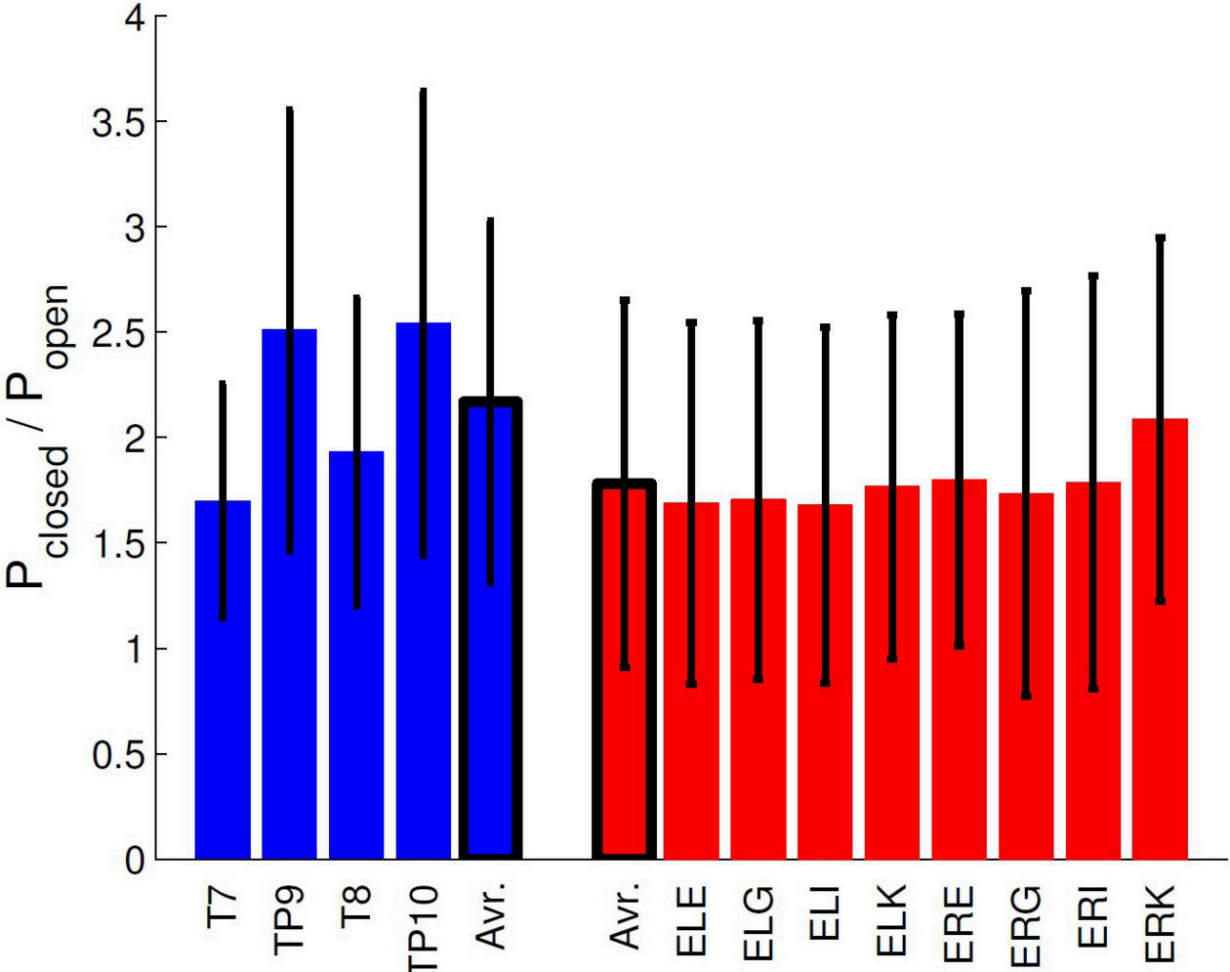
**Comparison of α-attenuation in scalp and ear-EEG electrodes, averaged over all subjects**. Errorbars show standard deviation.

Figure 8 (upper panel) shows the spectrogram for a single subject measurement during the α-attenuation paradigm, for ear-referenced data (to demonstrate that the difference is visible before cross-subject averaging), while the bottom panel shows the distribution of integrated power across subjects. Observe that the eyes open/eyes closed states are clearly distinguishable. A more rigorous quantification is given in Figure 9, where the average ratios of alpha power in the two states are shown for both scalp and ear channels. While the contrast is generally better on the scalp, the ear-EEG measurements are of a comparable quality.

## 4. Discussion

We observed that an ear-EEG electrode referenced to Cz is effectively identical to a conventional scalp electrode placed close to the ear and similarly referenced. This demonstrates that the surface potential acquired by the ear-EEG platform is of the same quality as that of conventional scalp electrodes.

Furthermore, the ASSR measurements demonstrate that the ear-EEG platform is well suited for studying the responses from primary auditory cortex, with ASSR powers lower, but comparable, to those measured with Cz-referenced temporal electrodes.

The analysis of ExA-referenced ERPs shows that the onset responses from the tone stimuli reproduce quite well, which was not the case for the MMN component. This is most likely due to the fact that the frontal sources of MMN are considerably further from the ear than primary auditory cortex (Tsolaki et al., 2015), and that the signal amplitude falls off as the inverse of the cube of the distance to the source, when electrode and reference are close together; this results in a very poor SNR for this signal in the ears.

We have also observed differences in the measured ERPs for right and left ear, when the local ExA-electrodes are used as references. We attribute this phenomenon to small anatomical differences between the hemispheres, which are magnified by the short electrode distances.

Finally, α-activity in resting and working states were compared, and as with the ASSR measurements, we found that the ear-EEG data had lower, but perfectly acceptable, discriminatory power compared to the conventional setup. Taken together, the positive ASSR and α-band results show that the ear-EEG platform is especially well suited for paradigms based on frequency-domain analysis.

Regarding possible problems with the ear-EEG setup, there are a couple of important comments to make:

- The positive results reported here are not due to any link between ear-EEG and scalp EEG. We say this with some certainty both because the two setups used different (but close) references and completely different grounds, but also because the effect of rereferencing to E*x*A after measurement, instead of having used E*x*A to begin with, was investigated as part of a pilot study (not included here). Finally, the results, in particular the ASSR-related ones, are entirely compatible with previously published work, Kidmose et al. (2013) in which ear-EEG and scalp EEG did not have close references.
- It is possible that the electrode gel on the canal electrodes (E*x*E,E*x*K,E*x*G,E*x*i) in some cases will have facilitated direct connections between neighboring canal electrodes. This can be a problem for the platform. However, as will be elaborated on in the conclusion, work is ongoing to drastically reduce the risk of this happening (through changing ear piece design, and switch from wet to dry electrodes). In the present study, it is important to notice that references are always placed outside the ear, to ensure that the measured signal is not particularly sensitive by the problem. As it stands, Figures 4–8 all serve to demonstrate that the measured signal is in fact dominated by products of neural processing, and not gel-based artifacts.

## 5. Conclusions and outlook

By referencing the ear electrodes to the scalp Cz electrode and comparing measurements to Cz-referenced temporal electrodes, we have demonstrated the feasibility of placing EEG electrodes inside the ear canal in such a way that both a reliable connection and a signal quality similar to that of a conventional scalp electrode can be maintained.

Using a classic oddball MMN paradigm we have found that the ear-EEG platform can detect ERPs from primary auditory cortex, but may have difficulties for sources further away from the ear.

Most interestingly, using both an ASSR and an α-attenuation paradigm, we have found that ear-EEG performs well in paradigms relying on frequency analysis. This is very promising, since it reinforces the primary aim of the ear-EEG platform—that it can be worn outside the laboratory, where oscillation studies are also more relevant.

Parallel to the work discussed in this paper, development of dry-contact electrode ear-EEG is ongoing, as well as experiments on alternative ear piece design and material. In this way, future ear-EEG pieces will have comfort levels comparable to custom fit hearing aids. Furthermore, work has been done to map the vulnerability of the platform toward physiological artifacts; here it has been found that the effect of jaw, head and eye movements were comparable for ear-EEG and scalp EEG (Kappel et al., 2014). All this, combined with the results presented here, validate realistic applications for the ear-EEG platform: (1) sleep monitoring, following on Koley and Dey (2012) who managed to successfully score sleep based only on EEG data from very few channels. Due to the potential comfort and ease of use of the ear-EEG platform, we envision that it could be highly useful in monitoring sleep quality outside of a clinical setting. (2) EEG micro state analysis. In the micro state literature it has been shown that a significant part of resting state EEG can be described as alternations between only four micro states (Britz et al., 2010), with different amounts of lateralization. Given the good correspondence between potentials measured using temporal and ear electrodes (Figure 2), we expect that ear electrodes referenced to the opposite ear should be able to describe much of the dynamics that have been uncovered using the micro state concept. In general, it would be interesting to test ear-EEG referenced to opposite ears in paradigms with a high degree of asymmetry between the hemispheres, as it seems that that would optimally combine the mobility of the platform with the large electrode distances of conventional scalp EEG.

### Conflict of interest statement

The authors declare that the research was conducted in the absence of any commercial or financial relationships that could be construed as a potential conflict of interest.

## Footnotes

^1^Danish: “De Videnskabsetiske Komitéer for Region Midtjylland,” case no: 1-10-72-48-15; and Danish: “Sundhedsstyrelsen,” case no: 2014080169.
